# Preventing deaths due to the hypertensive disorders of pregnancy

**DOI:** 10.1016/j.bpobgyn.2016.05.005

**Published:** 2016-10

**Authors:** Peter von Dadelszen, Laura A. Magee

**Affiliations:** a Institute of Cardiovascular and Cell Sciences, St George's University of London, UK; b Department of Obstetrics and Gynaecology, St George's University Hospitals NHS Foundation Trust, London, UK

**Keywords:** pregnancy hypertension, maternal mortality, societal interventions, medical interventions

## Abstract

In this chapter, taking a life cycle and both civil society and medically oriented approach, we will discuss the contribution of the hypertensive disorders of pregnancy (HDPs) to maternal, perinatal and newborn mortality and morbidity. Here we review various interventions and approaches to preventing deaths due to HDPs and discuss effectiveness, resource needs and long-term sustainability of the different approaches. Societal approaches, addressing sustainable development goals (SDGs) 2.2 (malnutrition), 3.7 (access to sexual and reproductive care), 3.8 (universal health coverage) and 3c (health workforce strengthening), are required to achieve SDGs 3.1 (maternal survival), 3.2 (perinatal survival) and 3.4 (reduced impact of non-communicable diseases (NCDs)). Medical solutions require greater clarity around the classification of the HDPs, increased frequency of effective antenatal visits, mandatory responses to the HDPs when encountered, prompt provision of life-saving interventions and sustained surveillance for NCD risk for women with a history of the HDPs.

## Highlights

• The contribution of pregnancy hypertension to maternal mortality is almost certainly under-estimated.
• Solutions relate to both societal and medical interventions.
• Improving the health, dietary and self-efficacy status of girls and adolescent women will reduce the health and societal impacts of pregnancy hypertension and its long-term health sequelae.
• The provision of effective, appropriately-resourced, and respectful maternity services will reduce the burden of adverse maternal and perinatal outcomes related to pregnancy hypertension.
• Appropriate interventions will reduce both the personal (for women) and inter-generational (for their children) impact of pregnancy hypertension on non-communicable disease risks and adverse outcomes.

## Introduction

The hypertensive disorders of pregnancy (HDPs), chronic (or pre-existing) hypertension, gestational hypertension and, especially, pre-eclampsia, remain leading causes of maternal and perinatal morbidity and mortality as well as identifying individuals at increased risk for premature cardiovascular disease *[1], [2].

## Diagnosis and classification

Here we use the Canadian definitions and diagnostic criteria for, and classification of, the HDPs [3].

Blood pressure (BP) should be measured at each antenatal visit. Hypertension in pregnancy is defined by a systolic BP (sBP) ≥140 mmHg and/or diastolic BP (dBP) ≥90 mmHg [3]. Severe hypertension is defined as sBP≥160 mmHg (instead of 170 mmHg) as that level of sBP reflects stroke risk *[3], [4], [5], [6], [7]. Elevated BP should be confirmed by repeat measurement, at least 15 min apart, being measured three times, the first value being disregarded and the average of the second and third taken as the BP value for the visit [3].

We suggest screening with a urinary dipstick at each antenatal visit. Proteinuria diagnosis can be performed on random samples (by a urinary dipstick or protein: creatinine ratio (PrCr)) or timed urine collections (usually 24 h). Quantification of urinary protein by 24-h urine collection is often inaccurate [8] and has been replaced by spot urine samples outside pregnancy [9]. A dipstick value of ≥++ proteinuria and PrCr of ≥30 g/mol represent significant proteinuria of 0.3 g/d, the current if flawed gold standard (SOGC 32); a threshold up to 40 g/mol may be more appropriate in multiple pregnancies (SOGC 33,34). Proteinuria should be quantified (by PrCr or 24-h urine collection) if pre-eclampsia is suspected, as dipstick proteinuria <++ has a significant false negative rate.

Chronic hypertension is defined as hypertension that predates pregnancy, or appears before 20 weeks or persists more than 3 months postpartum. It can be complicated by superimposed pre-eclampsia [3].

Gestational hypertension appears at ≥20 weeks and resolves by 3 months postpartum [3]. Associated risks depend on the gestational age at presentation and progression to pre-eclampsia; gestational hypertension at <34 weeks is associated with a ≈35% risk of pre-eclampsia, which takes an average of 5 weeks to develop [3].

Pre-eclampsia is the HDP associated with the greatest risks, particularly when it is severe or present at <34 weeks *[3], *[10], *[11]. The risk of small for gestational age (SGA) infants is primarily among women who present at <34 weeks, with macrosomia more common with the term pre-eclampsia [12]. Pre-eclampsia is most commonly defined by new-onset proteinuria and potentially other end-organ dysfunction. Table 1 outlines the end-organ dysfunction of pre-eclampsia: ‘adverse conditions’ and ‘severe complications’. ‘Adverse conditions’ consist of maternal symptoms, signs, abnormal laboratory results and abnormal foetal monitoring results that may herald development of severe maternal or foetal complications (including stillbirth). The ‘adverse conditions’ are those for which we wait and to which we respond (e.g., low oxygen saturation) to avoid the severe complications that we wish to avoid entirely (e.g., pulmonary oedema). Remember that a significant minority of women will present with unheralded eclampsia [13]. That response could be more intensive maternal or foetal monitoring, specific treatment or delivery. In the Canadian rubric, ‘severe complications’ of pre-eclampsia warrant delivery, irrespective of the gestational age [3].

It must be recalled that delivery does not ‘cure’ pre-eclampsia; it only initiates the recovery from the disease – and is the only intervention that will do so. Especially with early-onset pre-eclampsia, that arising before 34 weeks of gestation, many women experience a transient deterioration in their clinical state, with nadirs in platelet count and renal function and maximal liver enzyme abnormalities, before their recovery. Frequently, this period of clinical vulnerability may last up to 72 h postpartum *[1], [2]. In more developed countries, those women who die due to the consequences of pre-eclampsia almost uniformly do so postnatally.

## The global impact of pregnancy hypertension

### Maternal mortality

Complicating an estimated 3–10% of pregnancies *[1], [2], it has been estimated that the HDPs cause 30,000 maternal deaths annually [14]. However, verbal autopsy data from Pakistan imply that up to 40% of women who die from postpartum haemorrhage (PPH) complained of symptoms of pre-eclampsia (e.g., headache, visual disturbances and abdominal pain) or had seizures prior to the onset of their life-ending haemorrhage. It may be that a significant proportion of these women had pre-eclampsia that was complicated by disseminated intravascular coagulation, especially with the occurrence of abruption, but were never noted to be hypertensive by the formal health system. Such women would have bled sufficiently by the time of their presentation to mask their underlying hypertension; indeed, many will be hypotensive by the time they present for care. As PPH is estimated to cause 40,000 maternal deaths [14], the actual number of maternal deaths caused by the HDPs may be as high as 46,000 annually. Over 99% of these deaths occur in less-developed countries [15].

As with most causes of maternal death, avoidable delays in triage, transport and treatment contribute to a majority of HDP-related maternal deaths [16]. We believe that civil society has an important, and hitherto under-mobilised, role to play in overcoming these delays. This is an important theme to which we will return.

### Maternal morbidity

Approximately 30% of all maternal near-miss events will be due to pregnancy hypertension [17], [18], with near-miss events complicating about 420/100,000 deliveries [19] and approximately 230,000 HDP-related near-miss events per year (assuming 185 million pregnancies per annum).

### Stillbirth

Of the estimated 2.6 million stillbirths annually, approximately 16% occur in pregnancies complicated by pregnancy hypertension [20]. Of particular importance to the global health community is the fact that 11% of stillbirths are associated with pregnancies complicated by chronic hypertension, while only 5% are associated with pre-eclampsia (presenting as either pre-eclampsia or eclampsia) [20]. The global provision of effective antenatal surveillance could have a rapid impact on the association between chronic hypertension and stillbirth.

### Neonatal mortality

It has been estimated that the HDPs precede 10% of early neonatal deaths (8/1000 live births) [21] and a significant proportion of late neonatal deaths (3/1000 live births) [22]. This would approximate to about 1.5–2 million neonatal deaths annually.

### Neonatal morbidity

Due to their association with iatrogenic prematurity, foetal growth restriction and foetal overgrowth, the HDPs are identifiable risk factors for newborn morbidity (e.g., respiratory distress and neonatal hypoglycaemia) [23]. In addition, the rate of perinatal asphyxia, and resultant hypoxic-ischaemic encephalopathy, is increased due to the links between imperfect placentation and accelerated placental ageing with the HDP [24], [25]. Less recognised are the associations between maternal pre-eclampsia and neonatal thrombocytopoenia and neutropoenia [23]. The rates of neonatal morbidity will be inversely related to neonatal mortality rates, as survivability requires essential interventions that range from Kangaroo mother care to access to neonatal intensive care. As a rule of thumb, neonatal morbidities will occur 10–20 times more often than neonatal deaths.

## Civil society-based solutions

There is increasing recognition of the importance and value of preconception care [26]. Women and, all too frequently, girls enter pregnancy within the continuum of their individual life cycle. In our opinion, too often pregnancy has been focussed upon as a singular event that begins with either conception or a positive pregnancy test. If the global community wishes to achieve the sustainable development goal (SDG) 3.1, to reduce the global maternal mortality ratio to less than 70 per 100,000 live births by 2030 (http://www.un.org/sustainabledevelopment/health/), we need to intervene now to alter young girls' and women's health and societal status.

How can such changes be achieved in the context of pregnancy hypertension? Here we elucidate some possible solutions.

### Community engagement and mobilisation

Prost and colleagues have assessed the effects of women's groups practising participatory learning and action, compared with usual care, on birth outcomes in low-resource settings in Bangladesh, India, Malawi and Nepal [27]. Exposure to women's groups was associated with a 37% reduction in maternal mortality, a 23% reduction in neonatal mortality and a 9% trend towards a reduction in stillbirths. In a meta-regression analysis, the proportion of pregnant women in groups was linearly associated with reduction in both maternal and neonatal mortality, and if ≥30% of pregnant women participate in groups both maternal and neonatal mortality rates will fall (55% and 33%, respectively) in a cost-effective manner.

Since that review, Colbourn and colleagues evaluated a rural participatory women's group community intervention (CI) and a quality improvement intervention at health centres (FI) via a two-by-two factorial cluster randomised controlled trial (RCT) [28]. Following adjustment for clustering and stratification, the neonatal mortality rate was 22% lower in FI + CI than control clusters and the perinatal mortality rate was 16% lower in CI clusters. They were underpowered to assess maternal mortality.

Through the Community-Level Interventions for Pre-eclampsia (CLIP) cluster RCTs in Mozambique, Pakistan and India, we are testing the specific ability of HDP-oriented community engagement to improve maternal and foetal outcomes (http://www.thelancet.com/protocol-reviews/13PRT-9313). In the interim, and as pre-eclampsia and eclampsia can be detected in the community, we suggest that the non-specific benefits of community mobilisation are likely to improve HDP-related health outcomes. Such mobilisation could most usefully include gaining prior permissions to respond to maternal health emergencies for women without autonomy of decision-making, transport planning and micro-insurance initiatives to cover the facility's out-of-pocket expenses [29].

### Food security for girls and pregnant women

For women to be of adequate stature and be in good health prior to, and during, pregnancy they require food security from childhood – indeed, probably starting during foetal life and prior to their own conception. To achieve SDG 3.1 by 2030, we must provide food security to today's girls. Short stature (<164 cm) is associated with an approximate doubling of the risk for pre-eclampsia, especially severe disease [30], [31]. Short stature is observed significantly in women who were undernourished as girls [32]. Preferential feeding of male children and men, compared with girls and women, is prevalent in many less developed countries [33].

Women from a food-insecure household are three times as likely to be severely obese compared with women from food-secure households, after controlling for covariates: age, race, income, education, marital status and the number of children [34].

Once pregnant, women from food-insecure households gain 1.9 kg more than women from food-secure households [34]. In addition, Dean and colleagues have determined that maternal pre-pregnancy weight is a significant factor in the preconception period with being underweight contributing to a 32% higher risk of preterm birth, while obesity more than doubles the risk for pre-eclampsia and gestational diabetes [32]. Among nutrition-specific interventions, pre-conception folic acid supplements prevent 69% of recurrent neural tube defects, and multiple micronutrient supplements show promise of reducing the rates of congenital anomalies and risk of pre-eclampsia. While vitamin C and E supplementation does not decrease the risk of developing pre-eclampsia, adequate dietary intake of foods replete in antioxidants is associated with decreased rates of pre-eclampsia [35], [36], [37].

Both hypertension and pre-eclampsia are more common in pregnant girls and women who have insufficient intake of elemental calcium, an effect that is reversed with calcium replacement and supplementation [38], [39]. Indeed, in women with a history of severe pre-eclampsia, recent RCT data show that low-dose calcium replacement (500 mg/d to bring the total intake to 1 g/d) in women during pregnancy lowers the mean dBP (−2.6 mmHg) compared with women taking a matched placebo (+0.8 mmHg, mean difference −3.4 (95% CI −0.4 to −6.4), *p* = 0.025) [40].

Using cross-sectional data from India's third National Family Health Survey (NFHS-3, 2005–06), data were obtained from 39,657 women aged 15–49 years, who had had a live birth in the five years preceding the survey [41]. Women who had consumed either an adequately diversified diet or folate and iron supplements were approximately 35% less likely to report symptoms of either pre-eclampsia or eclampsia than women with inadequately diversified dietary intake. However, we recognise that personal recall of pre-eclampsia symptoms or diagnoses is vulnerable to recall bias.

In terms of perinatal outcomes, Bhutta and colleagues estimate that maternal undernutrition contributes to 800,000 neonatal deaths annually; stunting, wasting and micronutrient deficiencies are estimated to underlie nearly 3·1 million child deaths annually [42]. They conclude that continued investments in nutrition-specific interventions to avert maternal and child undernutrition and micronutrient deficiencies through community engagement and delivery strategies, including women's empowerment, agriculture, food systems, education, employment, social protection and social safety nets, can greatly accelerate progress in countries with the highest burden of maternal and child undernutrition and mortality.

### Delayed marriage and delayed first pregnancy

In many less developed country societies, marriage occurs early and is followed, in short order, by a first pregnancy. Both these events are modifiable through changes in civil societal expectations of girls and women.

Obstetric outcomes have been determined for teenage pregnancies in Finland, a country with a low teenage delivery rate and comprehensive high-quality antenatal care. In this privileged setting, teenagers face increased risks of several obstetric complications, including a trebling of the risk for developing eclampsia, while 13–15-year-olds have almost four times the odds for developing pre-eclampsia compared with women aged 20–24 years old [43]. This adverse influence of teenage pregnancy on maternal and perinatal outcomes is even stronger, compared with women in their twenties, in less-developed countries.

Kumar and colleagues have observed that the majority of pregnant teenagers are primigravid (83.2% vs. 41.4%) [44]. Gestational hypertension (11.4% vs. 2.2%), pre-eclampsia (4.3% vs. 0.6%) and eclampsia (4.9% vs. 0.6%) are more common in teenagers than in women in their twenties. In terms of perinatal risks, teenage mothers have pregnancies that are complicated more frequently by low birthweight (50.4% vs. 32.3%), premature delivery (51.8% vs. 17.5%), stillbirth (1.9% vs. 0.3%), neonatal morbidities (e.g., perinatal asphyxia (11.7% vs. 1.9%), jaundice (5.7% vs. 1.2%), respiratory distress (1.9% vs. 0.3%)) and neonatal mortality (3.8% vs. 0.5%). Relating back to the discussion about food insecurity and its contribution to obesity, the risks for a woman to develop either pre-eclampsia or eclampsia increase significantly with increasing body mass index (BMI) and decreasing age. Extremely obese teenagers are almost four times as likely to develop pre-eclampsia and eclampsia when compared with non-obese women aged 20–24 years of age [45].

There is convincing evidence that prior and prolonged exposure to paternal antigens may reduce the likelihood of pre-eclampsia [46]. Sexual and reproductive practices that minimise maternal exposure to paternal seminal fluid prior to pregnancy have been associated with an increased risk of pre-eclampsia. For example, women above the highest 10th percentile of vaginal exposure to paternal semen have a 70% reduced odds of developing pre-eclampsia compared with women below the lowest 25th exposure percentile [46]; these data are consistent with our growing understanding of the role of innate immunity in modulating trophoblast invasion during the first and second trimesters *[1], [2], [47].

### Birth spacing

From the literature on primarily more developed countries, it is well recognised that either a too short (<2 years) or too long (>10 years) birth interval increases a woman's risk for developing pre-eclampsia, especially recurrent disease [3]. In a less-developed country setting, De Jonge and colleagues analysed 5571 second- or higher-order deliveries in rural Bangladesh [48]. Younger women, women who deferred their fertility and those who achieved higher order parities were less likely to experience short birth intervals. Women who were socio-economically disadvantaged were more likely to experience a short birth interval, and a previous adverse outcome appeared to motivate women to delay a future pregnancy. Very short birth intervals of less than 21 months more than double the odds of either a stillbirth or newborn death. Therefore, giving women a reproductive choice is an important tool in reducing the HDP-related burden of adverse maternal and perinatal events.

### Facilities

Other important elements of societal responsibility to women whose pregnancies are complicated by the HDP are facility availability and enhancement. While community-based screening will identify women with hypertension, accessible and effective health facilities, staffed continuously by respectful and skilled health practitioners providing evidence-based care, are required to provide the essential life-saving interventions discussed in detail below. Commodity security for these diagnostic tools, medicines and emergency obstetric care provision is the responsibility of all levels of health systems, from local stock-taking through to regional and national governments [49].

Beyond the commodities required to modify disease-specific risks of stroke and eclampsia, facilities need to fulfil water, sanitation and hygiene (WASH) standards to provide safe care [50], especially as the only definitive method to initiate recovery from pre-eclampsia is delivery of the placenta [3]. This requires either labour induction or Caesarean delivery, the safety of both of which are WASH dependent. Campbell and colleagues have provided a carefully framed conceptual framework, linking WASH to improved maternal and reproductive health outcomes [51].

An example of how to mobilise funds for facility availability and enhancement is the experience of the Local Authority Transfer Fund in Kenya. This fund returns 5% of income tax to communities in a proportional manner, which has improved both accountability and community autonomy. A consequence has been an improvement in government income tax-based revenues, as communities see the return on their taxation being used to build such things as schools, health facilities and transport infrastructure [52]. Improvements across these infrastructures will lead to improved maternal and perinatal health outcomes.

## Medical solutions

### Health human resources

To support women during and after pregnancy, as well as their newborns, there needs to be an adequate health human resources infrastructure available to women wherever they live. Of the leading causes of maternal and perinatal mortality, the HDPs are those most amenable to a community-oriented approach as there is usually a lead time between the onset of symptoms and signs of pre-eclampsia and the evolution to life-threatening and life-ending complications.

Within communities, in CLIP, we are taking the approach of task-shifting the screening, initial diagnosis and initiation of lifesaving therapies to mobile health (mHealth)-supported community healthcare providers in women's communities (http://www.thelancet.com/protocol-reviews/13PRT-9313). These workers are agentes polivalentes elementares, lady health workers, and both accredited social health activists and auxiliary nurse midwives in Mozambique, Pakistan and India, respectively. An element of the mHealth-directed response to the detection of hypertension is either urgent (within 4 h) or non-urgent (within 24 h) transfer to referral facilities that provide comprehensive emergency obstetric care; the mHealth app, pre-eclampsia integrated estimate of risk (PIERS) on the Move (POM), has specific thresholds to guide women to seek urgent, rather than non-urgent, care (refs). However, it is important to recognise the increasing burden being placed on such workers through task-shifting in many countries – if this cadre of workers is to remain effective and accept more tasks, it requires an expansion in numbers and adequate support through pre-deployment training and ongoing professional development and supervision.

Once women reach health posts, primary health centres and inpatient facilities, they require access to trained and effective nurses, midwives, physician assistants and doctors. For maternity care, the global call is for strengthening and expansion of the midwifery workforce [53]. It is incumbent on whoever provides maternal and perinatal healthcare to do so respectfully, utilising the best evidence-based approach that local resources permit [54], [55]. Improving work environments will increase the likelihood of women receiving respectful care [56].

In all settings and at all levels, maternity care providers are obliged to undertake continuous professional development (CPD) tailored to their educational and skill levels – and national professional societies and ministries are obliged to provide CPD and the time to attend CPD courses.

### Screening for HDP risk

Approximately 1% of women live with chronic hypertension when they conceive, but at a global level, most of them are unaware of that. Therefore, routine antenatal BP measurement will identify hypertensive women, of whom many will be shown to have chronic hypertension as they remain hypertensive beyond 6 weeks postpartum.

The strongest clinical markers of pre-eclampsia risk identifiable at antenatal booking are recommended for screening for pre-eclampsia in the community [3]. Of the nine clinical predictors of pre-eclampsia among nulliparous women carrying singleton pregnancies, one is protective (miscarriage at ≤10 weeks with the same partner) and eight increase risks (younger maternal age, higher BP, higher BMI, family history of pre-eclampsia or coronary heart disease, woman with lower birthweight themselves, vaginal bleeding during early pregnancy and short duration of sexual relationship). Half of the women destined to develop pre-eclampsia would be detected using the model [3]. First-trimester uterine artery Doppler shows promise but needs further ‘real-life’ evaluation [3].

Second- and third-trimester markers of pre-eclampsia risk include measures of placental perfusion, vascular resistance and morphology; maternal cardiac output and systemic vascular resistance; foetoplacental unit endocrinology; maternal renal function; maternal endothelial function and endothelial–platelet interaction, oxidative stress, circulating angiogenic factors and glycosylated fibronectin *[1], [2], *[3], [47], [57]. Other than direct clinical measurements of BP and proteinuria, most of these tests are limited to well-resourced settings. As no single test predicts pre-eclampsia with sufficient accuracy to be clinically useful *[1], [2], *[3], [58], interest has grown in researching multivariable models that include clinical and laboratory predictors available at booking and thereafter *[1], [2], *[3], [59]. We await the results of definitive studies.

### Prevention of pre-eclampsia

#### Calcium replacement and supplementation

Lassi and colleagues have systematically reviewed calcium supplementation (1–2 g/d) for the prevention of pre-eclampsia and determined that calcium supplementation significantly reduces the risk of pre-eclampsia by 55% and by 64% in women with a low dietary intake of calcium [60]. In addition, calcium supplements have a significant protective effect on maternal mortality and serious morbidity and preterm birth, with trends to lowering risks for low birthweight and stillbirth.

Since this review, Hofmeyr and colleagues have reviewed the RCTs of calcium replacement (<1 g/d) rather than supplementation (1–2 g/d) [39]. Pre-eclampsia was reduced consistently by an average 62% with replacement, with or without co-supplements. The overall results were consistent with the single-quality trial of calcium replacement alone. Of note, one small trial of 60 women observed a statistical trend of >90% reduction in the miscarriage risk for women taking calcium replacement combined with antioxidants commencing at 8–12 weeks. These limited data are consistent with calcium replacement reducing the risk of pre-eclampsia. Currently, Professor Hofmeyr is conducting the calcium and pre-eclampsia trial, assessing the ability of pre-pregnancy calcium replacement to prevent recurrent pre-eclampsia (http://www.thelancet.com/protocol-reviews/11PRT-4028). Establishing a lower dose would have significant implications for the current guidelines and their global implementation.

#### Antiplatelet agents (low-dose aspirin) for prevention of pre-eclampsia

Results of the Lassi review show that antiplatelet agents are associated with a consistent reduction in pre-eclampsia (17% reduction)and preterm births <34 weeks (8% reduction) and a 14% reduction for all types of perinatal deaths (foetal, neonatal, infant) [60]. We recommend that antiplatelet agents should be given to pregnant women at a high risk of pre-eclampsia or those with gestational hypertension, since they lead to reduction of vast adverse outcomes of pregnancy for both the mother and the newborn.

### Provision of antenatal care

While access to both antihypertensives and magnesium sulphate clearly matters, in our opinion the greatest health system priority to improve maternal and perinatal outcomes is the provision of effective maternity care. The World Health Organisation four-visit model is associated with increased risks of perinatal loss [61], and this probably relates to the gap in antenatal surveillance between 36 weeks and delivery. Most HDPs present or deteriorate either near or at term. The accelerating pattern of antenatal visits (four-weekly visits until 28 weeks, fortnightly to 36 weeks and weekly thereafter) was designed in Edinburgh largely for the identification of pre-eclampsia to afford the opportunity for timely delivery [62]. Since the first triennial report of the UK Confidential Enquiries into Maternal Deaths (1952–54), pre-eclampsia- and eclampsia-related deaths have fallen by approximately 90% [63]. Over 90% of that fall was achieved prior to the introduction of either effective antihypertensives for the management of pregnancy hypertension [64] or the use in the UK of magnesium sulphate for eclampsia prevention and treatment following the Collaborative Eclampsia and Magpie Trials, respectively [65], [66]. Once a diagnosis has been made, then an adequate health system can respond through the provision of inpatient care, ongoing surveillance, BP control, seizure prophylaxis and timed delivery. Similar data exist for Sri Lanka following the introduction of comprehensive maternity care, including vital registration, registration of midwives, prenatal coverage (health centre- and home-based) and facility strengthening [67]. Therefore, personalised risk assessment through antenatal care is an effective tool for reducing adverse pregnancy outcomes, especially when included in a package of health-service enhancements.

### Detection of the HDP

#### Blood pressure

BP measurement remains a cornerstone of the screening for, and diagnosis of, pre-eclampsia *[3], [63], *[68]. An important advance in the move toward offering every pregnant woman accurate BP measurement and detection of pregnancy hypertension has been the development of the semi-automated Microlife BP 3AS1-2 sphygmomanometer®, which has been validated in women with the hypertension of pre-eclampsia [69]. This device avoids the errors intrinsic in manual BP measurement in pregnancy (especially terminal digit preference [70], can be used by minimally trained community-level healthcare providers and costs less than $25 USD per unit.

#### Urinary protein measurement

The obstetric gold standard of 24-h urine collection is frequently inaccurate and not a precise measure of either proteinuria or creatinine clearance [8]. Additionally, a recent systematic review of the spot PrCr and albumin: creatinine (ACR) ratios as diagnostic tests for significant proteinuria (compared with 24-h collection) in hypertensive pregnant women determined that the spot PrCr ratio (cut-off point, 30 g/mol) is a reasonable “rule-out” test for detecting significant proteinuria of 0.3 g/day or more in hypertensive pregnancy [71]. Practitioners using either 24-h collections and/or the PrCr ratio in pregnancy must be particularly aware of the interaction between the protein estimation method and urinary concentration, as the pyrocatechol violet-dye method is vulnerable to overestimating urinary protein in dilute urine [63]. Information related to the use of the ACR in pregnant women remains insufficient to guide practice [63]. In pregnancy, urine is often dilute, and we found that almost 25% of ACR results are clinically uninformative [72].

Within the PIERS cohort, dipstick proteinuria performs as well (or poorly) as other methods of assessing proteinuria for prediction of adverse outcomes [73]. Our findings support the view that once significant proteinuria has been identified (by dipstick [≥++], PrCr ratio or 24-h collection), further proteinuria assessment should not be used to guide clinical decision-making related to either short- or long-term maternal prognosis. Rather, the serum creatinine value should be used to monitor renal function and risk in women with pre-eclampsia [11]. In settings without laboratory support, the heaviness of dipstick proteinuria is predictive of adverse perinatal, but not maternal outcomes [10].

### Time-of-disease maternal risk assessment

Not all women with pregnancy hypertension are destined to either die or suffer permanent sequelae. Therefore, our PIERS initiative has been an investigating tool to provide personalised risk assessment and medicine to women who have a diagnosis of a HDP. In the studies summarised below, the outcome of interest was maternal mortality or other serious complications of pre-eclampsia [11].

We have assessed the performance of maternal symptoms, proteinuria, oxygen saturation by pulse oximetry (SpO_2_), uric acid, platelet counts and liver tests individually to identify those hypertensive pregnant women who are more likely to suffer severe complications [73], [74], [75], [76], [77], [78]. Of these, only SpO_2_ showed any capacity as a test that can be used in isolation, including predicting non-cardiorespiratory adverse maternal outcomes (area under the receiver-operator characteristic (AUC ROC) >0.7) [77]. Therefore, we deemed that multivariable models were required.

#### Resource-constrained settings

We developed and validated the demographics-, symptom- and sign-based miniPIERS risk prediction model to provide a simple, evidence-based tool to identify pregnant women in resource-constrained settings at increased risk of death or major hypertensive-related complications [10]. Developed and validated on 2081 women with any HDP admitted to a participating centre in Brazil, Fiji, Pakistan, South Africa and Uganda, the final miniPIERS model included parity (nulliparous vs. multiparous), gestational age on admission, headache/visual disturbances, chest pain/dyspnoea, vaginal bleeding with abdominal pain, sBP and dipstick proteinuria. The miniPIERS model was well calibrated and had an area under the receiver operating characteristic curve (AUC ROC) of 0.77. External validation AUC ROC using the fullPIERS cohort (below) was 0.71. A predicted probability ≥25% to define a positive test classified women with 85.5% accuracy. Our conclusion is that miniPIERS can be used to identify women who would benefit most from interventions such as magnesium sulphate, antihypertensives or transportation to a higher level of care.

To strengthen miniPIERS, we used South African and Pakistani data to recalibrate and extend miniPIERS to include SpO_2_ [79]. Women with SpO_2_ < 93% had a 30-fold increase in risk of adverse maternal outcome compared with those with SpO2 > 97%. After recalibration and extension, the miniPIERS model including SpO_2_ (vs. not including SpO_2_) had improved sensitivity (32.8% vs. 49.6%) at the cost of minimally decreased specificity (91.5% vs. 96.2%).

In the CLIP trials, we are testing the performance of miniPIERS, and other thresholds that mandate an urgent response, using country-specific versions of the POM app [80], [81]. The POM app provides culturally sensitive, easy-to-use guidance for community healthcare providers. In rural Sindh, for example, a lady health worker is guided through antenatal screening, BP measurement and, if the pregnant woman is hypertensive, a range of responses that can include lady health worker-administered magnesium sulphate (for women at high risk) and, if the woman is severely hypertensive, oral methyldopa, prior to urgent referral for definitive facility-based care. If the woman is assessed as having less risk, then she is advised to seek facility-based care within 24 h.

#### Well-resourced settings

We developed and internally validated the fullPIERS model with the aim of identifying the risk of fatal or life-threatening complications in women with pre-eclampsia within 48 h of hospital admission to tertiary obstetric centres in Australia, Canada, New Zealand and the UK [11]. Independent predictors of adverse maternal outcome included in fullPIERS are gestational age, chest pain or dyspnoea, SpO_2_, platelet count and serum creatinine and aspartate transaminase concentrations. FullPIERS predicted adverse maternal outcomes within 48 h of study eligibility (AUC ROC 0·88) and performed well (AUC ROC >0·7) up to 7 days after eligibility. In addition, we reassessed the performance of fullPIERS using predictor variables obtained within 6 and 24 h of admission and found that the stratification capacity, calibration ability and classification accuracy of the model remained high [82].

Akkermans and colleagues performed an external validation of fullPIERS using prospectively collected data from two tertiary care obstetric centres that participated in the Pre-eclampsia Trial Amsterdam (PETRA) [83] and observed that fullPIERS predicted adverse maternal outcomes within 48 h (AUC ROC 0.97) and up to 7 d after inclusion (AUC ROC 0.80). More recently, in prospective external validation cohort in India, women with a >30% predicted probability were 17.5 times more likely to experience adverse maternal outcome, implying that fullPIERS has particular strength as a “rule-in” test [84]. Further external and temporal validation of fullPIERS is underway, and a fullPIERS mHealth app is being developed.

### Time-of-disease foetal risk assessment

#### Resource-constrained settings

The primary method for detection of the at-risk foetus has been maternal awareness of decreased foetal movement. Winje and colleagues have performed a systematic review of randomised and NRSs assessing the effects of interventions to enhance maternal awareness of decreased foetal movement [85]. Results were not pooled due to substantial heterogeneity between studies. Three RCTs and five NRSs assessed the effects of interventions on stillbirth and perinatal death. RCT data are inconclusive for the prevention of stillbirth. All NRSs favoured intervention over standard care; three studies reported significant while two reported non-significant reductions in stillbirth or perinatal deaths. Therefore, on balance, the evidence supports awareness of foetal movement being beneficial.

In addition to foetal movements, within the PIERS initiative, we have developed and internally validated a prognostic model for perinatal death that could guide community-based antenatal care of women with a HDP in low-resourced settings as part of an mhealth application [86]. Of the 1688 women admitted ≥32^+0^ weeks' gestation with a HDP from the five low-resourced countries in the miniPIERS cohort, 110 (6.5%) suffered a perinatal death. A logistic regression-derived model to predict perinatal death included maternal age, a count of symptoms (0, 1 or ≥2) and dipstick proteinuria. The AUC ROC is 0.75. The model, which excluded foetal movements due to unavailability of that variable, correctly identified 42/110 (38.2%) additional cases as high risk (probability >15%) of perinatal death compared with the use of only gestational age.

#### Well-resourced settings

Most experts suggest that the use of non-stress tests/cardiotocography (NST/CTG) be restricted to high-risk groups only, including women with pre-eclampsia [12]. However, evidence is lacking to either support this practice or guide the practitioner on the frequency of testing. Most authorities suggest that women with mild pre-eclampsia undergo weekly NST/CTG, whereas those with severe early-onset pre-eclampsia (in particular, in the presence of foetal growth restriction or oligohydramnios) be followed at least twice weekly [12]. The authors of a recent Cochrane review concluded that ‘there is almost no evidence to date to indicate an optimal antenatal surveillance method for infants identified with impaired growth’ [87], highlighting the urgent need for research in this area.

In the context of both pre-eclampsia and intrauterine growth restriction (IUGR), the use of the biophysical profile tends to falsely reassure, leading to an excess of adverse perinatal events [88], [89], [90].

Doppler velocimetry can be used in the assessment of foetuses from high-risk pregnancies, including IUGR and pre-eclampsia (particularly early-onset disease). Umbilical-artery Doppler assessment in high-risk pregnancies is associated with a 29% reduction in perinatal mortality, as well as 10% fewer inductions of labour and Caesarean deliveries [12]. The combination of abnormal middle cerebral artery peak systolic velocity and reversed flow in the ductus venosus increases the risk of perinatal mortality by 10-fold [12].

At this time, the available evidence suggests that, in cases of pre-eclampsia associated with IUGR, integrating Doppler, NST/CTG and fluid assessment may help further define the risk of perinatal death and assist in determining the optimal timing of delivery [12]. However, as there is no outcome prediction model that integrates and weights the independent value of each of these parameters, we believe that research to develop and validate a fullPIERS-like model for high-risk foetuses is required.

### Treatments

#### Non-severe hypertension

BP-pressure targets for women with non-severe hypertension during pregnancy have been much debated. The Control of Hypertension in Pregnancy Study (CHIPS) trial was an open, international, multicentre RCT [91]. CHIPS recruited 987 women at 14^+0^–33^+6^ weeks, who had non-proteinuric chronic (74.6%) or gestational (25.4%) hypertension, an office dBP of 90–105 mmHg (or 85–105 mmHg if taking antihypertensive medications) and a live foetus, to receive either less-tight (target dBP 100 mmHg) or tight (target dBP 85 mmHg) control. The rates of pregnancy loss or high-level neonatal care >48 h during the first 28 postnatal days did not differ between the less-tight and tight control groups (31.4% and 30.7%, respectively) and neither did maternal complications (3.7% and 2.0%, respectively), despite a mean dBP that was higher in the less-tight control group by 4.6 mm Hg. Severe hypertension (≥160/110 mm Hg), a threshold for stroke risk, developed in 40.6% of the women in the less-tight control group and in 27.5% of the women in the tight-control group – a difference that is highly significant, both statistically and, for women, clinically.

In CHIPS, methyldopa and labetalol were used commonly both at randomisation (24.6% and 24.6%, respectively) and post randomisation (22.8% and 44.1%, respectively). Following adjusted analyses, methyldopa (vs. labetalol) at randomisation was associated with fewer babies with a birthweight <10th centile. Methyldopa (vs. labetalol) post randomisation was associated with fewer CHIPS primary outcomes, birthweight <10th centile, severe hypertension, pre-eclampsia and delivery before both 34 and 37 weeks [92].

While CHIPS was limited to non-proteinuric women with hypertension, we believe that the results are relevant to women with pre-eclampsia. On balance, we believe that tight control is the preferred response for all types of pregnancy hypertension, especially in resource-constrained settings where access to follow up and surveillance is more limited. This approach is not deleterious to the foetus, adds maternal safety by keeping women away from severe hypertension and is consistent with approaches outside pregnancy. Methyldopa appears to be the preferable agent due to its effects, presence of essential medicines lists [93] and, for under-resourced settings, cost; the other options are listed in Table 2. Note that angiotensin-converting enzyme inhibitors (e.g., captopril) and angiotensin-2 receptor blockers (e.g., lisinopril) should not be used due to the increased risk of stillbirth with their use *[3], [64].

#### Severe hypertension

A sBP ≥160 mmHg and/or dBP ≥110 mmHg requires a response, with immediate targets being to lower BP to under those thresholds within hours *[1], *[3], [64], *[68]. Severe systolic hypertension is an independent risk factor for stroke in pregnancy [4], [5], [6], [7]. The UK ‘Confidential Enquiries into Maternal Deaths’ has identified failure to recognise the severity of and to treat the severe (particularly systolic) hypertension of pre-eclampsia as the single most serious failing in the clinical care of those women who died from strokes [6], [7].

Such acute elevations in BP may reflect either the development or progression of pre-eclampsia and should precipitate detailed clinical, laboratory and ultrasound assessment of the pregnancy.

Our antihypertensive of choice for severe pregnancy hypertension is oral nifedipine (either capsule or intermediate-acting tablet) because it has a more reliable effect than both labetalol and hydralazine and less frequent occurrence of drug-induced hypotension [63]. Nifedipine is safe to use with concurrent magnesium sulphate [63]. Our preference is for oral agents in non-obtunded women as their use can be initiated by nurses/midwives rather than the common requirement for the doctor's presence for an intravenous agent; the presence of a doctor can be delayed because of concurrent activities such as Caesarean deliveries.

Within the context of the PRE-EMPT initiative, Gynuity Health Projects are conducting a RCT with a head-to-head comparison of three oral antihypertensives for severe pregnancy hypertension, nifedipine, labetalol and methyldopa (http://pre-empt.cfri.ca/treatment/treatmentgynuity-trial). The results of this trial will be pertinent to all caregivers.

As a rule of thumb, our approach is to reserve nifedipine for use in women with severe pregnancy hypertension and to use either labetalol and/or methyldopa for longer term medication to achieve synergism of effect and to use beta-blockade to avoid the acute cardiovascular risks associated with nifedipine capsules. In addition, if a woman experiences an acute elevation in her BP on, say, labetalol, it seems logical to us not to use more of that agent for acute BP control. Effective antihypertensives are available in all settings to manage severe pregnancy hypertension [63], [64] (Table 2).

#### Prevention and treatment of seizures

Magnesium sulphate is the first-line treatment of eclampsia and its prevention, especially in women with severe disease *[3], *[68]. We reviewed current regimens in less developed countries for magnesium sulphate [94]. The commonly used regimens are 14 g Pritchard loading dose (4 g iv + 10 g im), full Pritchard regimen (same loading dose + 5 g/4 h im maintenance), an im loading dose only regimen (10 g im; without the 4 g iv), the Zuspan regimen (4 g iv loading dose + 1 g/h iv) and the Sibai regimen (4 g iv loading dose + 2 g/h). Most regimens use additional 2 g iv/im if seizures occur after initiation of therapy. The Zuspan regimen then increases the maintenance dose to 2 g/h.

In terms of task-shifting, a single RCT of community administration of a magnesium sulphate loading dose before referral to a facility versus limiting treatment to facilities reduced the occurrence of recurrent eclampsia by 78%. Due to concerns related to maternal safety, cost and resource availability, there has been an interest in alternative regimens for magnesium sulphate administration, particularly in less-developed countries; however, we do not support moving to abbreviated or lower dose courses until adequately powered and well-designed RCTs have been published [63], [94]. In addition, clarifying exactly who among women with non-severe pre-eclampsia requires magnesium sulphate, how much women should receive and for how long remains a research priority.

We believe that the continued use of benzodiazepines, phenytoin or lytic cocktail to be dangerous and requiring robust and sustained efforts by national societies and health administrators to be halted.

#### Foetal neuroprotection

In addition to its use for eclampsia prophylaxis and treatment, in women with pre-existing or gestational hypertension, magnesium sulphate should be considered as a cost-effective therapy to decrease the risk of cerebral palsy (and ‘death or cerebral palsy’) in the setting of ‘imminent preterm birth’ (within the next 24 h) up to 33^+6^ weeks [95], [96]. We advocate using the same regimen for foetal neuroprotection as clinicians use for eclampsia prevention and treatment. This will reduce dosing errors and strengthen confidence in magnesium sulphate use.

Magnesium sulphate for foetal neuroprotection should not be used concurrently with tocolysis. Its use should be limited to when the clinician is certain of early preterm birth – the loading dose alone appears to be effective.

#### Antenatal corticosteroids

When administered at ≤34^+6^ weeks, antenatal corticosteroids accelerate foetal pulmonary maturity and decrease neonatal mortality and morbidity, including women with HDPs [3]. RCTs that administered steroids at 33^+0^–34^+6^ weeks resulted in reduced neonatal RDS [3], a subject of ongoing trials. The beneficial effects of steroids can be observed when the first dose is administered as late as within 4 h before birth. As long as dating is secure, there is no evidence of short- or long-term maternal or foetal adverse effects of a single course of antenatal corticosteroids. Inaccurate dating and the use of corticosteroids close to term may cause harm [97].

If expectantly managed, women with pre-eclampsia remote from term (usually <34^+0^ weeks) will deliver within two weeks of corticosteroid administration, but the duration of pregnancy prolongation varies from hours to weeks. All eligible women with pre-eclampsia should receive antenatal corticosteroids. If women with pre-eclampsia remain pregnant seven or more days after their initial course, then a single, and only a single, rescue course of corticosteroids appears to be warranted [98].

#### Fluids

Routine preloading with a fixed volume of crystalloid (i.e., 500–1000 mL) will not prevent BP falls in normal women prior to Caesarean delivery [3]; no specific studies exist for HDPs. Preloading may increase the risk of life-threatening pulmonary oedema [3]. Hypotension should be treated with vasopressors as an infusion or small boluses [3].

Oliguria (<15 mL/h) is common in pre-eclampsia, particularly postpartum. In the absence of pre-existing renal disease or a rising creatinine, oliguria should be tolerated over hours, to avoid volume-dependent pulmonary oedema [3]. Fluid balance should be closely monitored and frusemide limited to pulmonary oedema treatment [3].

#### When to deliver

In response to the new Canadian definition of severe pre-eclampsia, any woman with severe pre-eclampsia should deliver *[3], [63]. The manner of that delivery will be determined by the woman's gestational age and other obstetric factors.

##### At term

Women at term (≥36^+0^ weeks) with either pre-eclampsia or non-proteinuric gestational hypertension are best managed by a policy of induction of labour *[3], [63]. Among women randomised ≥37^+0^ weeks, for every seven labour inductions, there was one fewer Caesarean delivery. Women with chronic hypertension appear to benefit most from induction at 38^+0^–39^+6^ weeks [99].

##### Near term

However, a policy of induction near term (34^+0^–36^+6^ weeks) is associated with no measurable benefit for mothers, but with threefold greater risks of newborn respiratory distress [3].

##### Remote from term

Remote from term, RCT, case-control and cohort data support a policy of expectant (vs. interventionist) care [63]. Generally, about half of the women will remain undelivered 48 h after presentation (timeframe for antenatal corticosteroid administration and effect) and be eligible for expectant management. In RCTs, the in utero time gained from eligibility to delivery is about 14 days, whereas in non-RCT studies, the time gained is generally 7–10 days.

Expectant management should only be attempted in institutions experienced in the management of pre-eclampsia. Failure to provide obsessive surveillance during expectant management is a life-threatening gap in care [63]. For women who present before 24 weeks' gestation (or local limits of viability), expectant management is unlikely to offer any perinatal advantages while maternal risks persist and rise [63]. For such women with pre-viability pre-eclampsia, the offer of pregnancy termination for maternal indications should be made. This remains an option denied to women in many societies where full reproductive choice is withheld.

When a decision is made to pursue delivery at term gestational ages, vaginal delivery is a viable option if the maternal and foetal conditions allow it, although Caesarean delivery rates are significant (14–19%) [100]. At lower gestational ages, rates of successful vaginal delivery are better than might be anticipated: 69% at 32^+0^–33^+6^ weeks and 48% at 28^+0^–31^+6^ weeks. However, vaginal delivery is rarely achieved at less than 28 weeks (7%) [100].

### Long-term health and non-communicable diseases (NCDs): addressing the SDGs between generations

#### The mothers

Women who develop a HDP, deliver preterm and have a baby with the lowest quintile of birthweight acquire similar risk for premature cardiovascular disease as women who smoke [101].

Any weight gain between pregnancies predicts pre-eclampsia and other pregnancy complications [3]. Observational data suggest that in women who are morbidly obese, bariatric surgery lowers rates of subsequent HDP [3]. Women with pre-existing hypertension should receive recommended cardiovascular risk factor screening and treatment [3].

As pregnancy is a biological ‘stress test’ of sorts, women with a prior HDP (particularly associated with preterm delivery or adverse perinatal outcome) should be informed of their increased future health risks, including hypertension, cardiovascular and cerebrovascular morbidity and mortality, subsequent renal disease, thromboembolism, hypothyroidism and type 2 diabetes mellitus [3]. Barriers to compliance with a healthy diet and lifestyle include poor postpartum physical and psychological recovery and lack of postpartum medical and psychological support from healthcare providers [3]. These barriers need to be addressed in a way that is both culturally responsive and effective.

#### The offspring

The increasingly robust evidence for developmental origins for health and disease is of particular importance as the world moves to embrace the SGDs. Approximately 20% of a person's risk for premature cardiovascular disease is determined by birth. Girls who are born small due to the modifiable sociocultural factors discussed above are more likely to have pregnancies complicated by HDPs as well as premature cardiovascular disease. Interventions across the spectrum are likely to create a virtuous cycle that will accelerate progress towards the NCD elements of the SDGs.

Pre-eclampsia is a robust predictor of cardiovascular and reproductive health in the offspring of those pregnancies [3].

D Effects of maternal hypertension and its therapies on child neurobehavioral development.

Superimposed pre-eclampsia (vs. chronic hypertension alone) has no adverse effect on (or slightly better) intellectual development of the offspring; however, there is no information about the antihypertensives received by the mothers during pregnancy [3]. For children born SGA, neurodevelopmental outcomes are improved if the mother develops pregnancy hypertension [102].

HDPs, and their treatment with antihypertensives, may predict generally modest long-term effects on child development; clinicians should be aware of these mild disabilities and be prepared for early school-age interventions. For example, in one study, methyldopa (but not labetalol) may be associated with lower IQ, the duration of treatment being an independent negative predictor of children's performance IQ [3].

**Practice points**

Communities need to mobilise around the topics of pregnancy and its complications.
Women, and not girls, need to enter pregnancy when they are well nourished and at a time of their choice.
Women require access to fully staffed and well-resourced health facilities.
Women should be screened for the HDPs, including pre-disease screening of risk.
Women with an HDP should receive personalised time-of-disease risk assessment.
All women with severe pregnancy hypertension should receive antihypertensive therapy, whether pregnant or postpartum.
All women with non-severe pregnancy hypertension should have their BP normalised.
Magnesium sulphate is the agent of choice for the prevention and treatment of eclampsia as well as for foetal neuroprotection.
Decisions about delivery are dependent on gestational age.
Vaginal delivery should always be considered.
Women with any HDP, and their offspring, are at increased risk for premature cardiovascular disease.

**Research agenda**

Population-level estimates of the incidence and impact of the HDPs on pregnancies.
Populations' and decision-makers’ understanding of the HDPs to aid in identifying barriers and facilitators for future interventions.
Evaluation of the use of biomarkers, such as placental growth factor and glycosylated fibronectin, to screen populations of pregnant women at increased risk for pre-eclampsia and other placental complications of pregnancy in those requiring the Edinburgh model of antenatal care and those for whom the four-visit model will suffice.
Identification of optimal antihypertensive to use in the setting of severe pregnancy hypertension will reduce the avoidable burden of HDP-related strokes.
Adequately powered and well-designed studies of both reduced-dosing regimens and community administration of magnesium sulphate.
Optimal timing of delivery for pregnant women with chronic hypertension.

## Conflict of interest statement

Laura Magee and Peter von Dadelszen own shares in LionsGate Technologies, manufacturer of a mobile pulse oximeter. No other potential conflicts are known.

## Figures and Tables

**Table 1:** Defining the adverse features and severe complications of pre-eclampsia (modified from Magee *et al* Preg Htn).

Organ system affected	Adverse conditions (that increase the risk of severe complications)	Severe complications (that warrant delivery)
Central nervous system	○ Headache and/or visual symptoms	○ Eclampsia ○ PRES ○ Cortical blindness or retinal detachment ○ Glasgow coma scale <13 ○ Stroke, TIA, or RIND
Cardiorespiratory	○ Chest pain and/or dyspnoea ○ Oxygen saturation <97%	○ Uncontrolled severe hypertension (over a period of 12 h despite use of three antihypertensive agents) ○ Oxygen saturation <90%, need for ≥50% oxygen for >1 h, intubation (other than for Caesarean section) ○ Pulmonary oedema ○ Positive inotropic support ○ Myocardial ischaemia or infarction
Haematological	○ Elevated WBC count ○ Elevated INR or aPTT ○ Low platelet count	○ Platelet count <50×10^9^/L ○ Transfusion of any blood product
Renal	○ Elevated serum creatinine ○ Elevated serum uric acid	○ Acute kidney injury (creatinine >150 μM with no prior renal disease) ○ New indication for dialysis
Hepatic	○ Nausea or vomiting ○ RUQ or epigastric pain ○ Elevated serum AST, ALT, LDH, or bilirubin ○ Low plasma albumin	○ Hepatic dysfunction (INR >2 in absence of DIC or warfarin) ○ Hepatic haematoma or rupture
Foetoplacental	○ Non-reassuring FHR ○ IUGR ○ Oligohydramnios ○ Absent or reversed end-diastolic flow by Doppler velocimetry	○ Abruption with evidence of maternal or foetal compromise ○ Reverse ductus venosus A wave ○ Stillbirth

AST – aspartate transaminase, ALT – alanine transaminase, aPTT – activated partial thromboplastin time, DIC – disseminated intravascular coagulation, FHR – foetal heart rate, LDH – lactate dehydrogenase, INR – international normalised ratio, PRES – posterior reversible leukoencephalopathy syndrome, RIND – reversible neurological deficit 24–48 h, RUQ – right upper quadrant, TIA – transient ischaemic attack ≤24 h, WBC – white blood cell.

**Table 2:** Commonly used agents for severe and non-severe pregnancy hypertension (modified from PvD CHR).

Indication/Agent	Dosage	Onset	Peak	Duration	Comments
Severe hypertension					
Hydralazine	Start with 5 mg iv; repeat 5–10 mg iv every 3 min, or 0.5–10 mg/h iv, to a maximum of 20 mg iv (or 30 mg im)	5 min	30 min	2–4 h	May increase the risk of maternal hypotension. Appears less effective than nifedipine but more effective than labetalol.
Labetalol iv	Start with 20 mg iv; repeat 20–80 mg iv every 30 min, or 1–2 mg/min (then switch to oral (max 300 mg))	5 min	30 min	4 h	Best avoided in women with asthma or heart failure. Neonatology should be informed if the woman is in labour, as parenteral labetalol may cause neonatal bradycardia.
Labetalol po	200 mg loading dose; repeat further 200 mg doses every 45 min (then switch to regular oral (max 1200 mg/d))	20 min–2 h	1–4 h	8–12 h (dose-dependent)	
Nicardipine iv	Initial infusion rate 2.5–5 mg/h, increasing by 2.5 mg/h every 5 min (max 15mg/h)	5 min	20 min	4–6 h	Currently not recommended as a first-line agent by any national or international guideline committee
Nifedipine capsule	5–10 mg capsule to be swallowed or bitten then swallowed every 30 min	5–10 min	30 min	≈6 h	Be aware of the distinction between short-acting nifedipine capsules, the intermediate-acting tablets and the slow-release tablets. Avoid nifedipine capsules in women with known coronary artery disease, severe aortic stenosis and pre-existing diabetes of ≥15 y duration due to risks of acute coronary syndromes.
Nifedipine intermediate-acting/PA	10 mg tablet; repeat 10 mg doses every 45 min (then switch to regular oral medication) (max 120 mg/d)	30 min	4 h	12 h	
Non-severe hypertension					
Methyldopa	250–500 mg po bid-qid (max 2 g/d)	40 min	3–6 h	12–24 h	There is no evidence to support a loading dose of methyldopa. CHIPS data suggest that methyldopa is preferable to labetalol. There are reassuring neurodevelopmental data for methyldopa.
Labetalol	100–400 mg po bid-tid (max 1200 mg/d)	20 min–2 h	1–4h	8–12 h (dose-dependent)	Best avoided in women with asthma or heart failure. Some experts recommend a starting dose of 200 mg po bid.
Nifedipine intermediate-acting/PA	10-40 mg tablet po bid-tid (max 120 mg/d)	30 min	4 h	12 h	Be aware of the distinction between short-acting nifedipine capsules, the intermediate-acting tablets and the slow-release tablets
Nifedipine XL preparation	20–60 mg po OD (max 120 mg/d))	60 min	6 h	24 h	
